# Genotyping and Drug Resistance Profile of Clinical Isolates of *Candida albicans* from Vulvovaginal Candidiasis in the Eastern China

**DOI:** 10.1007/s11046-022-00616-x

**Published:** 2022-01-24

**Authors:** Nan Hong, Yan Lei, Huan Chen, Xiaofei Chen, Kin Ming Tsui, Danyang Hu, Wanqing Liao, Liang Yan, Hong Zhang, Rongfen Zhao, Gang Wu, Nong Yu, Shuwen Deng

**Affiliations:** 1 Department of Medical Microbiology, The People’s Hospital of Suzhou New District, Suzhou, Jiangsu China; 2 Department of Dermatology, South China Hospital, Health Science Center, Shenzhen University, Shenzhen, China; 3 Department of Dermatology, Jinling Hospital, Nanjing University School of Medicine, Nanjing, China; 4 Department of Dermatology, Peking Union Medical College Hospital, Chinese Academy of Medical Sciences and Peking Union Medical College, Beijing, China; 5 Division of Infectious Diseases, Faculty of Medicine, University of British Columbia, Vancouver, Canada; 6 Department of Pathology, Sidra Medicine, Doha, Qatar; 7 Shanghai Key Laboratory of Medical Molecular Mycology, Department of Dermatology, Changzheng Hospital, Second Military Medical University, Shanghai, China; 8 Department of Dermatology, PLA General Hospital of Central Theater Command, Wuhan, China; 9 Nanjing Chia Tai Tianqing Pharmaceutical Co., Ltd, Nanjing, China

**Keywords:** Genetic diversity, Antifungal susceptibility, Resistance, *Candida albicans*, Vulvovaginal candidiasis, China

## Abstract

A total of 244 *Candida albicans* isolates recovered from vulvovaginal candidiasis (VVC) patients in Suzhou, Eastern China, were investigated. According to CLSI documents M27-A4 and M59-3ed/M60-2ed, the MIC geometric means of nine antifungals in increasing order were micafungin (0.048 mg/L), anidulafungin (0.132 mg/L), caspofungin (0.19 mg/L), itraconazole (0.23 mg/L), posaconazole (0.25 mg/L), voriconazole (0.28 mg/L), 5-flucytosine (0.44 mg/L), amphotericin B (0.49 mg/L) and fluconazole (2.01 mg/L) respectively. Of note, 6.5% (16/244) *C. albicans* isolates showed resistance mainly to anidulafungin (mono-echinocandin resistance), while voriconazole had the lowest susceptibility rate of 34.8% (85/244), followed by fluconazole 59.4% (145/244), respectively. All isolates were genotyped by allelic combination of 3 microsatellite markers (CEF3, CAIII and LOC4). A total of 129 different allelic genotypes were identified, in which seven different clades were recognized with a discriminatory power of 0.96. Genotypes A-D were present in 35% of the isolates. In conclusion, decrease in antifungal drug susceptibility to *C. albicans* isolates from VVC is alarming. Our findings revealed the genetic diversity of *C. albicans* isolates among VVC patients and provided insights into the molecular epidemiology of *Candida* infections in China.

**Supplementary Information:**

The online version contains supplementary material available at 10.1007/s11046-022-00616-x.

## Introduction

Vulvovaginal candidiasis (VVC) is one of the most common vaginitis caused by *Candida albicans*, which accounts for 80–95% of all episodes of VVC worldwide, though the number of non-*albicans* species (such as *Candida glabrata*) as etiological agents of VVC is increasing [1, 2]. An increasing prevalence of fungal resistance is also reported in antifungal surveillance studies globally [3–5]. Antifungal susceptibility testing of *C. albicans* isolates therefore plays a crucial role for appropriate and effective management strategies of VVC [5]. In addition, it is important to look into the population genetic structure and epidemiology of *C. albicans* infections because of increasing reports indicating dynamic changes in the antifungal susceptibility profile with different genotypes and geographic origins [6]. Thus, understanding the genetic diversity of *C. albicans* could help clinicians to implement appropriate diagnostic, therapeutic and preventive strategies [7, 8]. Microsatellite analysis is highly reproducible with strong discriminatory power and is widely applied in molecular typing of fungal pathogens [9]. In previous studies, microsatellite analysis has proved to be a powerful tool in investigating the relationship between genetic diversity and antifungal susceptibility of *C. albicans* isolates [10, 11]. However, knowledge on the population genetic structure and antifungal susceptibility profile of isolates from VVC patients in China still remains limited. Therefore, in this study, we investigated the antifungal susceptibility profile on hundreds of *C. albicans* isolates from VVC patients in eastern China (Suzhou area) based on M27-A4 and M59/M60 documents approved in 2020 for interpretive breakpoints and epidemiological cutoff (ECV). Also, we performed molecular typing utilizing three microsatellite loci (CAIII, CEF3, and LOC4) to investigate the genetic variability.

## Materials and Methods

### Isolates and Identification

A total of 244 vaginal *C. albicans* isolates were recovered from patients with vulvovaginal candidiasis in the People's hospital of Suzhou New District during Jan to Dec. 2018. The patients and case definition, vaginal samples collecting information have been reported in previous publication [5]. All isolates were identified to the species level by sequencing D1/D2 domain of 26S ribosomal DNA gene as described previously [5]. Isolates information and GenBank accession numbers of D1/D2 sequences are listed in supplementary table 1.

### Microsatellite Analysis

Microsatellite genotyping was performed with all 244 *C. albicans* isolates, based on a panel of three different short-nucleotide repeat fragments, using fluorescently labeled primers CAIII (5′-Tamra -TTGGAATCACTTCACCAGGA-3′, 5′-TTTCCGTGGCATCAGTATCA-3′); CEF3 (5′- Hex-TTTCCTCTTCCTTTCATATAGAA-3′, 5′- GGATTCACTAGCAGCAGACA-3′); LOC4 (5′- FAM –GTAATGATTACGGCAATGAC-3′, 5′-AGAACGACGTGTACTATTGG-3′) [11]. A multiplex polymerase chain reaction (PCR) was performed in 10 μl reaction volumes containing 5 μl of Qiagen Multiplex PCR (2x, Lot 148031955), 0.25 μl of each primer (forward and reverse), 3 μl of ddH_2_O, and 1 μl of genomic DNA. PCR amplifications were performed in a thermocycler (BOECO, TC-Pro, Germany) operating with a temperature-cycling program that consisted of an initial denaturing step at 95 °C for 15 min, followed by 35 cycles of 30 s at 94 °C, 90 s at 57 °C, and 60 s at 72 °C, with a final extension step of 10 min at 72 °C. The size of the fragments was determined by addition of the GeneScan LIZ500 marker and subsequent analysis on the Applied Biosystems 3730 DNA analyzer. Assignment of repeat numbers in each marker was determined from the GeneScan data by using the Peak GeneMapper 5.0 software (Applied Biosystems, Foster City, CA, USA). Allele-sharing distance matrices were generated from the tandem repeat numbers and were used as input for UPGMA clustering analysis. The UPGMA clustering of the 244 *C. albicans* isolates was performed using R package phangorn. The UPGMA tree was then plotted using R (version 3.4.4). The discrimination power (DP) of the microsatellite genotyping method used in this study was calculated by the online calculator created by the university of the basque country (http://insilico.ehu.es/mini_tools/discriminatory_power) which evaluates the probability of any pair of isolates to belong to distinct genotypes.

### Antifungal Susceptibility Testing

All isolates were tested for in vitro susceptibility to nine antifungal drugs agents according to the CLSI reference guideline M27-A4 [12]. Antifungal drugs tested were anidulafungin (ANF), caspofungin (CAS), micafungin (MFG), amphotericin B (AmB), 5-flucytosine (5-FC), fluconazole (FLC), itraconazole (ITR), voriconazole (VRC) and posaconazole (POS). Anidulafungin and voriconazole were purchased from Toronto Research Chemicals Inc (Canada), micafungin was provided by Astellas Pharma (Japan), and remaining antifungals were obtained from Sigma-Aldrich. *Candida parapsilosis* ATCC 22019 and *Candida krusei* ATCC 6258 were used as control strains in all experiments. All isolates were sub-cultured onto Sabouraud Dextrose Agar at 35 °C for 24 h for viability and purity. Colonies were suspended in sterile saline, and the final inoculum concentration of the suspension was adjusted to 0.5–2.5 × 10^3^ CFU/mL with RPMI1640 broth medium. The 96-well plates were incubated for 24 or 48 h at 35 °C, and the minimum inhibitory concentrations (MIC) were determined visually. Drug concentration ranges, time of MIC readings and interpretive breakpoints used for 9 antifungal agents are listed in Supplementary table 2.

### Interpretation of MIC Results

Interpretation of susceptibility was performed by applying the clinical breakpoints (CBPs) defined by the document M60-2ed [13]. In the absence of CBPs, isolates were defined as having a wild-type (WT) or a non-wild-type (NWT) drug susceptibility phenotype (to amphotericin B, posaconazole, itraconazole and 5-flucytosine) according to the epidemiological cutoff values (ECV) defined by the document M59-3ed [14], as shown in Supplementary table 2.

### Ethical Statement

Ethical approval and patient consensus were not considered necessary due to the descriptive nature of the study that implied only the samples obtained during routine laboratory activity.

## Results

Table 1 summarized the antifungal susceptibility profile of 244 *C. albicans* isolates to 9 antifungals. The MIC geometric means of the antifungals across all isolates were the following (in increasing order): micafungin (0.048 mg/L), anidulafungin (0.132 mg/L), caspofungin (0.19 mg/L), itraconazole (0.23 mg/L), posaconazole (0.25 mg/L), voriconazole (0.28 mg/L), 5-flucytosine (0.44 mg/L), amphotericine B (0.49 mg/L) and fluconazole (2.01 mg/L).

**Table 1:** Antifungal susceptibility profile of 244 *C. albicans* isolates from VVC to 9 antifungal agents

Drug	Range	MIC_50_/MIC _90_	GM	S (%)	I (%)	SDD (%)	R (%)	NWT (%)
MFG	0.016–1	0.0313/0.25	0.048	227/93.0%	15/6.2%	NA	2/0.8%	NA
ANF	0.016–4	0.125/0.5	0.132	210/86.1%	18/7.4%	NA	16/6.5%	NA
CAS	0.0313–1	0.25/0.25	0.191	222/91.0%	21/8.6%	NA	1/0.4%	NA
FLC	0.125–64	2/16	2.011	145/59.4%	NA	36/14.8%	63/25.8%	NA
VRC	0.125–4	0.25/1	0.278	85/34.8%	126/51.6%	NA	33/13.5%	NA
POS	0.0313–4	0.25/1	0.251	NA^a^	NA	NA	NA	221/90.5%
ITR	0.0313–16	0.25/1	0.232	NA	NA	NA	NA	NA
AmB	0.25–1	0.5/0.5	0.485	NA	NA	NA	NA	0/0%
5-FC	0.125–64	0.25/2	0.436	NA	NA	NA	NA	NA

a, not applicable; GM, geometric mean values; S%, susceptible rate; I%, intermediate susceptible rate; SDD%, susceptible dose dependent rate; R%, resistant rate; NWT%, rate of non-wild type

Table 2 summarized MIC distribution, resistance rate and NWT rate of 244 *C. albicans* isolates from VVC to 9 antifungal agents. Of the 244 *C. albicans* isolates, 86% (210) were susceptible to the three echinocandins tested, and 6.5% of the *C. albicans* isolates were resistant to anidulafungin which was much higher than resistance rates obtained for the two other echinocandins tested (0.4% and 0.8%, respectively).

**Table 2:** MIC distribution, R%, NWT% of 244 *C. albicans* isolates from VVC to 9 antifungal agents

Drug	0.016	0.0313	0.0625	0.125	0.25	0.5	1	2	4	8	16	32	≥ 64	R%	NWT%
MFG	16.4	61.5^a^	80.7	87.3	93.0^b^	99.2	100.0	100.0	100.0	100.0	100.0	100.0	100.0	0.8%	38.50%
ANF	3.3	8.2	37.3	67.6	86.1	93.4	97.1	99.2	100.0	100.0	100.0	100.0	100.0	6.5%	32.4%
CAS	0.0	2.9	10.7	34.4	91.0	99.2	100.0	100.0	100.0	100.0	100.0	100.0	100.0	0.4%	NA
FLC	0.0	0.0	0.0	5.7	13.9	25.4	42.6	59.4	74.2	86.1	92.2	99.6	100.0	25.8%	74.6%
VRC	0.0	0.0	0.0	34.8	66.0	86.5	98.0	99.2	100.0	100.0	100.0	100.0	100.0	13.5%	100%
POS	0.0	5.3	23.0	42.2	59.4	81.6	90.2	97.5	100.0	100.0	100.0	100.0	100.0	NA^c^	90.5%
ITR	0.0	14.8	24.2	39.8	60.2	84.4	93.0	95.9	98.8	99.6	100.0	100.0	100.0	NA	NA
AmB	0.0	0.0	0.0	0.0	11.5	93.0	100.0	100.0	100.0	100.0	100.0	100.0	100.0	NA	0
5-FC	0.0	0.0	0.0	35.7	51.6	66.0	76.2	95.5	98.0	98.8	99.2	98.2	100.0	NA	NA

a, percentage of isolates with MIC ≦ ECV; b, percentage of isolates with MIC ≦≦ susceptible CBP; NA, not applicable; R%, resistant rate; NWT%, rate of non-wild type

The in vitro activity of triazoles against 244 isolates of *C. albicans* was variable. Fluconazole was active against 59.4% (145/244) of the isolates tested, and 25.8% (63/244) of the *C. albicans* isolates were resistant to fluconazole. However, 14.8% (36/244) of the isolates were fluconazole SDD. Voriconazole had reduced activity to approximately more than half of the isolates with a susceptibility rate of 34.8% (85/244), whereas 51.6% (126/244) of the isolates exhibited an intermediate susceptibility and 13.5% (33/244) were resistant to voriconazole. Resistance to both fluconazole and voriconazole was found in 23 isolates of *C. albicans*. However, 90.5% of the *C. albicans* isolates showed relatively higher MICs than epidemiological cutoff value (ECV 0.06 mg/L) to posaconazole. Regarding the susceptibility to itraconazole, 60.2% of the isolates had MIC values > 0.125 mg/L, and 15.6% had MIC values > 0.5 mg/L.

As expected, all *C. albicans* isolates tested revealed lower MICs than ECV (2 mg/L) to amphotericin B, while 98.0% of isolates showed MIC lower than 4 mg/L to 5-flucytosine.

**Fig. 1:**
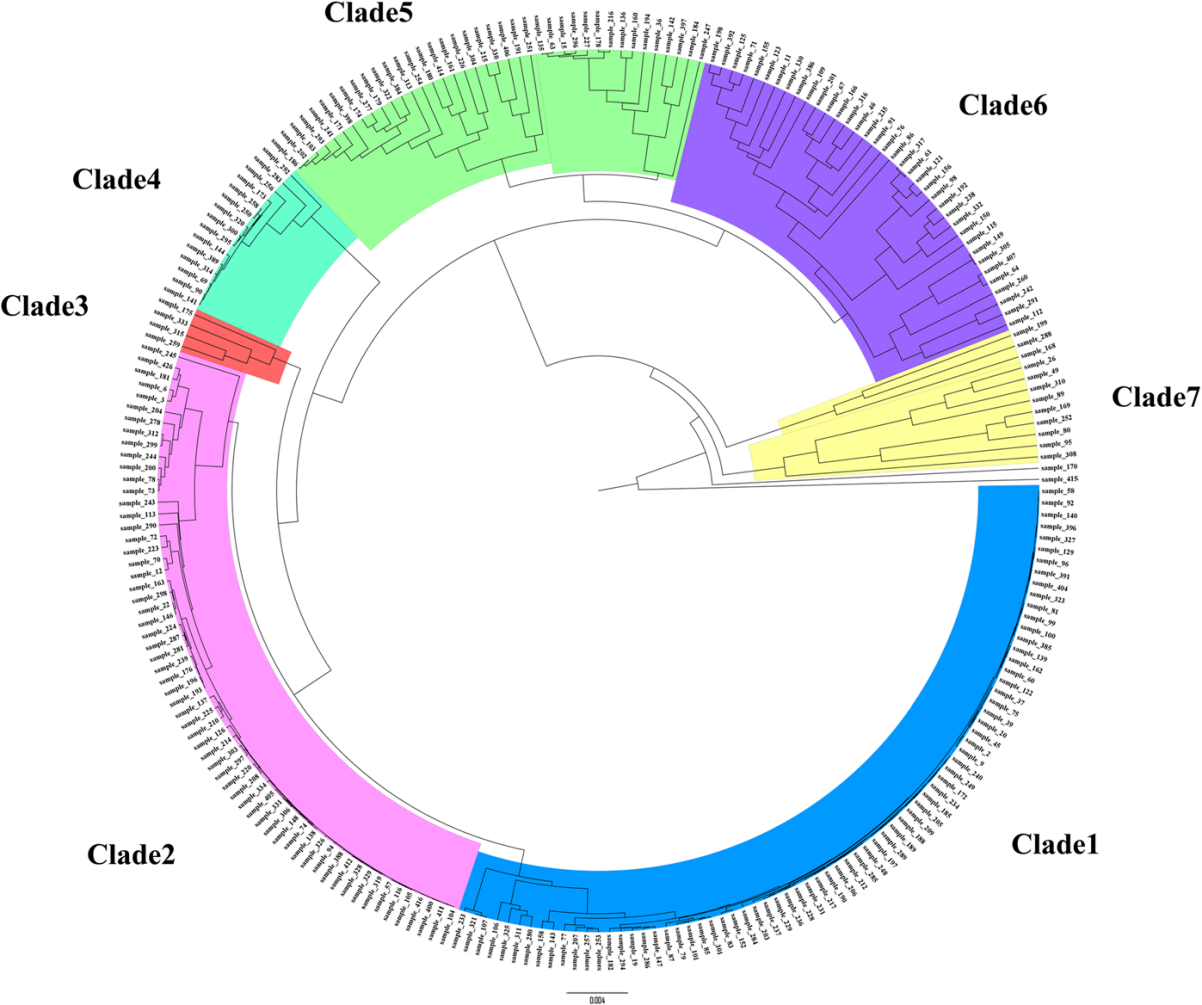
UPGMA clustering dendrogram of the 244 *C. albicans* isolates from VVC patients based on the combined analysis of CEF3, CAIII and LOC4 microsatellite markers

The genotypic relationship of 244 *C. albicans* isolates from VVC patients was determined based on UPGMA analysis of 3 microsatellite markers CEF3, CAIII, LOC4. Seven clades were recognized (Fig. 1). Microsatellite genotyping of three loci showed considerable diversity among 244 *C. albicans* isolates, and 129 different allelic combinations were identified among 244 unrelated *C. albicans* isolates with 108 singleton genotypes (Supplementary table 3). The combined discriminatory power (DP) of the 3-loci (CAIII, CEF3, and LOC4) typing method was 0.96. The most frequent genotype was genotype A (34/244, 13.9%) followed by genotypes B (25/244, 10%) and C (18/244, 7%). The other remaining genotypes had a frequency lower than 5% (Supplementary table 1).

## Discussion

This study investigated the antifungal susceptibility profiles and genetic diversity of 244 *C. albicans* isolates from VVC patients in Suzhou, eastern China.

The majority of these isolates showed good antifungal activity to the three echinocandins. Micafungin (MIC_50_/MIC_90_: 0.031/0.25 mg/L) showed slightly higher potency than caspofungin (MIC_50_/MIC_90_: 0.25/0.25 mg/L) and anidulafungin (MIC_50_/MIC_90_: 0.125/0.5 mg/L) which was consistent with previous studies [5, 15]. Of note, less than 10% of *C. albicans* isolates exhibited an intermediate susceptibility to the three echinocandins tested, whereas 16 out of the 244 (6.5%) *C. albicans* isolates showed resistance mainly to anidulafungin (mono-echinocandin resistance). Furthermore, of the 16 isolates of *C. albicans* with high-MIC anidulafungin phenotype (MIC ≧ 1 mg/L), only one isolate had MIC≧1 mg/L for micafungin, and another isolate had MIC ≧ 1 mg/L for caspofungin, which is not in agreement with previous reports indicating that *C. albicans* resistant to echinocandins accounted for less than < 1% [6, 16] and that the mono-echinocandin resistance phenotype was rare [17, 18]. However, our previous study reported one isolate with MIC 1 mg/L for anidulafungin among 207 *C. albicans* isolates from VVC patients in western China [5]. Pfaller et al*.* [19] presented similar results on anidulafungin against *C. albicans* causing invasive infections. Lindberg et al*.* [20] determined the in vitro susceptibility of *Candida* isolates from the blood samples of patients with candidemia at a Swedish hospital and found that 17% of *C. albicans* isolates were not susceptible to anidulafungin by applying the EUCAST CBPs. However, when the CLSI CBPs were applied, all the isolates exhibited susceptibility to anidulafungin. Thus, isolates with this high anidulafungin MIC warrant further study.

All triazoles tested had reduced activity against *C. albicans* isolates from VVC patients. Voriconazole had the lowest susceptibility rate of 34.8% (85/244), followed by fluconazole 59.4% (145/244), respectively. Of note, about half of *C. albicans* isolates (51.6%) tested were classified as exhibiting an intermediate susceptibility to voriconazole, whereas 25.8% and 13.5% of the isolates were resistant to fluconazole and voriconazole, respectively. Compared to our previous study in western China [5], fluconazole resistance of *C. albicans* isolates from VVC significantly increased in eastern China (8.2% resistance rate in western China versus 25.8% resistance rate in Suzhou, eastern China). Our results were also compatible with most Chinese reports indicating that approximately half of *C. albicans* isolates causing VVC were susceptible to fluconazole [21–23]. Notably, percentages of isolates with resistance and I/SDD to fluconazole and voriconazole in Suzhou were much higher than those in previous reports from Boikov et al. [15], Ying et al. [21] and Shi et al. [23].

The MIC values for posaconazole in present study were high with 90.5% NWT isolates which was higher than 60% NWT isolates found in our previous study in west area when the CLSI ECV (0.06 mg/L) was applied. Our findings are conflicting with those from North America [24] and Kuwait [25] which showed good activity of posaconazole against *C. albicans* isolates from VVC (MIC_90_: 0.03 mg/L and 0.064 mg/L, respectively). However, 37.6% NWT isolates to posaconazole were reported from invasive candidiasis [26]. Although there was no interpretive breakpoint for itraconazole to *C. albicans* based on the newly described CLSI breakpoints [13, 14], our findings are unusual since 60% isolates had MIC value > 0.125 mg/L, and 15.6% (38/244) isolates had MIC value ≧ 1 mg/L (Table 2). Overall, resistance to triazoles among *C. albicans* isolates was found to be increasing over time, and this could be associated with frequent usage of these azoles in clinical settings in Suzhou area [27], Therefore, a continued surveillance on the antifungal susceptibility among *C. albicans* is necessary to guide treatment of VVC.

Microsatellite genotyping of three loci showed considerably high diversity, and 129 different allelic combinations were identified among 244 isolates of *C. albicans* recovered from patients with VVC. Seven clades were recognizable based on a categorical analysis of CEF3, CAIII, LOC4 microsatellite markers in combination with UPGMA clustering (Fig. 1). We observed that the genotypes A-C (77/244) accounted for 31.5% of the isolates. Moreover, a total of 108 isolates were shown to represent unique molecular types which account for 44% of the isolates tested, indicating high genetic diversity within the isolates in this study (Supplementary table 1). Our results confirmed the high genetic diversity among *C. albicans* isolates reported in previous similar studies [10, 11]. Sharifynia et al*.* [11] analyzed the different allelic combinations of 3 microsatellite loci (CAIII, CEF3, and LOC) in 105 independent *C. albicans* strains isolated from patients in Iran, and identified 93 unique microsatellite genotypes that clustered into six clades. Garcia-Hermoso et al*.* [28] identified 38 different genotypes among 50 *C. albicans* strains in a surgical intensive care unit using the microsatellite analysis of EF3, CDC3 and HIS3 loci. The relatively high genetic variability among *C. albicans* samples may be related to high dynamism of the *C. albicans* genome with recombination, rearrangement and rapid adaptation to host and drug resistance [29].

## Conclusions

Drug resistances of *C. albicans* present significant challenges to implement appropriate therapies and treatment for VVC. Therefore, antifungal susceptibility testing of *Candida* isolates plays a crucial role in the management of *Candida* infections. The microsatellite data of *C. albicans* confirmed that this medically important yeast has maintained high levels of genetic variability in Eastern China.

## Supplementary Information

Below is the link to the electronic supplementary material.

Supplementary file1 (DOCX 45 kb)

Supplementary file2 (DOCX 17 kb)

Supplementary file3 (DOCX 53 kb)

## Data Availability

Sequence data from this study are available in Genbank database under the accession number MZ172462-MZ172702 and MZ226435-MZ226437.
