# Supplementary material for: Genotyping and Drug Resistance Profile of Clinical Isolates of Candida albicans from Vulvovaginal Candidiasis in the Eastern China
Source: Mycopathologia. 2022 Jan 24;187(2-3):217–24. doi: 10.1007/s11046-022-00616-x (PMC9124162; doi:10.1007/s11046-022-00616-x)
Supplement: Supplementary file 1 — Supplementary file1 (DOCX 45 kb) [file 11046_2022_616_MOESM1_ESM.docx]

| Isolate Nr. | Species Name | Source | Origin | GenBank accession Nr. | Genotype |
| --- | --- | --- | --- | --- | --- |
| 122 | *Candida albicans* | Suzhou | Vaginal secretion | MZ172476 | A |
| 129 | *Candida albicans* | Suzhou | Vaginal secretion | MZ172480 | A |
| 139 | *Candida albicans* | Suzhou | Vaginal secretion | MZ172487 | A |
| 140 | *Candida albicans* | Suzhou | Vaginal secretion | MZ172488 | A |
| 162 | *Candida albicans* | Suzhou | Vaginal secretion | MZ172505 | A |
| 190 | *Candida albicans* | Suzhou | Vaginal secretion | MZ172527 | A |
| 2 | *Candida albicans* | Suzhou | Vaginal secretion | MZ172608 | A |
| 20 | *Candida albicans* | Suzhou | Vaginal secretion | MZ172546 | A |
| 206 | *Candida albicans* | Suzhou | Vaginal secretion | MZ172542 | A |
| 212 | *Candida albicans* | Suzhou | Vaginal secretion | MZ172548 | A |
| 217 | *Candida albicans* | Suzhou | Vaginal secretion | MZ172552 | A |
| 229 | *Candida albicans* | Suzhou | Vaginal secretion | MZ172569 | A |
| 231 | *Candida albicans* | Suzhou | Vaginal secretion | MZ172561 | A |
| 248 | *Candida albicans* | Suzhou | Vaginal secretion | MZ172577 | A |
| 284 | *Candida albicans* | Suzhou | Vaginal secretion | MZ172593 | A |
| 289 | *Candida albicans* | Suzhou | Vaginal secretion | MZ172598 | A |
| 323 | *Candida albicans* | Suzhou | Vaginal secretion | MZ172629 | A |
| 327 | *Candida albicans* | Suzhou | Vaginal secretion | MZ172632 | A |
| 37 | *Candida albicans* | Suzhou | Vaginal secretion | MZ172640 | A |
| 385 | *Candida albicans* | Suzhou | Vaginal secretion | MZ172642 | A |
| 39 | *Candida albicans* | Suzhou | Vaginal secretion | MZ172646 | A |
| 391 | *Candida albicans* | Suzhou | Vaginal secretion | MZ172652 | A |
| 396 | *Candida albicans* | Suzhou | Vaginal secretion | MZ172648 | A |
| 404 | *Candida albicans* | Suzhou | Vaginal secretion | MZ172654 | A |
| 45 | *Candida albicans* | Suzhou | Vaginal secretion | MZ172664 | A |
| 58 | *Candida albicans* | Suzhou | Vaginal secretion | MZ172669 | A |
| 60 | *Candida albicans* | Suzhou | Vaginal secretion | MZ172670 | A |
| 75 | *Candida albicans* | Suzhou | Vaginal secretion | MZ172682 | A |
| 81 | *Candida albicans* | Suzhou | Vaginal secretion | MZ172688 | A |
| 9 | *Candida albicans* | Suzhou | Vaginal secretion | MZ172702 | A |
| 92 | *Candida albicans* | Suzhou | Vaginal secretion | MZ172696 | A |
| 96 | *Candida albicans* | Suzhou | Vaginal secretion | MZ172699 | A |
| 99 | *Candida albicans* | Suzhou | Vaginal secretion | MZ172701 | A |
| 100 | *Candida albicans* | Suzhou | Vaginal secretion | MZ172462 | A |
| 104 | *Candida albicans* | Suzhou | Vaginal secretion | MZ172465 | B |
| 105 | *Candida albicans* | Suzhou | Vaginal secretion | MZ172466 | B |
| 116 | *Candida albicans* | Suzhou | Vaginal secretion | MZ172473 | B |
| 138 | *Candida albicans* | Suzhou | Vaginal secretion | MZ172486 | B |
| 148 | *Candida albicans* | Suzhou | Vaginal secretion | MZ172495 | B |
| 208 | *Candida albicans* | Suzhou | Vaginal secretion | MZ172544 | B |
| 220 | *Candida albicans* | Suzhou | Vaginal secretion | MZ172553 | B |
| 297 | *Candida albicans* | Suzhou | Vaginal secretion | MZ172606 | B |
| 303 | *Candida albicans* | Suzhou | Vaginal secretion | MZ172612 | B |
| 306 | *Candida albicans* | Suzhou | Vaginal secretion | MZ172615 | B |
| 319 | *Candida albicans* | Suzhou | Vaginal secretion | MZ172625 | B |
| 326 | *Candida albicans* | Suzhou | Vaginal secretion | MZ172631 | B |
| 328 | *Candida albicans* | Suzhou | Vaginal secretion | MZ172633 | B |
| 329 | *Candida albicans* | Suzhou | Vaginal secretion | MZ172634 | B |
| 331 | *Candida albicans* | Suzhou | Vaginal secretion | MZ172636 | B |
| 334 | *Candida albicans* | Suzhou | Vaginal secretion | MZ172639 | B |
| 388 | *Candida albicans* | Suzhou | Vaginal secretion | MZ172644 | B |
| 400 | *Candida albicans* | Suzhou | Vaginal secretion | MZ172651 | B |
| 405 | *Candida albicans* | Suzhou | Vaginal secretion | MZ172655 | B |
| 411 | *Candida albicans* | Suzhou | Vaginal secretion | MZ172658 | B |
| 412 | *Candida albicans* | Suzhou | Vaginal secretion | MZ172659 | B |
| 416 | *Candida albicans* | Suzhou | Vaginal secretion | MZ172662 | B |
| 57 | *Candida albicans* | Suzhou | Vaginal secretion | MZ172668 | B |
| 74 | *Candida albicans* | Suzhou | Vaginal secretion | MZ172681 | B |
| 94 | *Candida albicans* | Suzhou | Vaginal secretion | MZ172697 | B |
| 101 | *Candida albicans* | Suzhou | Vaginal secretion | MZ172463 | C |
| 172 | *Candida albicans* | Suzhou | Vaginal secretion | MZ172512 | C |
| 182 | *Candida albicans* | Suzhou | Vaginal secretion | MZ172521 | C |
| 185 | *Candida albicans* | Suzhou | Vaginal secretion | MZ172523 | C |
| 188 | *Candida albicans* | Suzhou | Vaginal secretion | MZ172525 | C |
| 189 | *Candida albicans* | Suzhou | Vaginal secretion | MZ172526 | C |
| 197 | *Candida albicans* | Suzhou | Vaginal secretion | MZ172533 | C |
| 205 | *Candida albicans* | Suzhou | Vaginal secretion | MZ172541 | C |
| 209 | *Candida albicans* | Suzhou | Vaginal secretion | MZ172545 | C |
| 234 | *Candida albicans* | Suzhou | Vaginal secretion | MZ172563 | C |
| 236 | *Candida albicans* | Suzhou | Vaginal secretion | MZ172565 | C |
| 237 | *Candida albicans* | Suzhou | Vaginal secretion | MZ172566 | C |
| 240 | *Candida albicans* | Suzhou | Vaginal secretion | MZ172570 | C |
| 249 | *Candida albicans* | Suzhou | Vaginal secretion | MZ172578 | C |
| 285 | *Candida albicans* | Suzhou | Vaginal secretion | MZ172594 | C |
| 301 | *Candida albicans* | Suzhou | Vaginal secretion | MZ172611 | C |
| 79 | *Candida albicans* | Suzhou | Vaginal secretion | MZ172686 | C |
| 85 | *Candida albicans* | Suzhou | Vaginal secretion | MZ172690 | C |
| 146 | *Candida albicans* | Suzhou | Vaginal secretion | MZ172493 | D |
| 163 | *Candida albicans* | Suzhou | Vaginal secretion | MZ172506 | D |
| 176 | *Candida albicans* | Suzhou | Vaginal secretion | MZ172516 | D |
| 193 | *Candida albicans* | Suzhou | Vaginal secretion | MZ172530 | D |
| 196 | *Candida albicans* | Suzhou | Vaginal secretion | MZ172532 | D |
| 22 | *Candida albicans* | Suzhou | Vaginal secretion | MZ172560 | D |
| 224 | *Candida albicans* | Suzhou | Vaginal secretion | MZ172555 | D |
| 281 | *Candida albicans* | Suzhou | Vaginal secretion | MZ172591 | D |
| 287 | *Candida albicans* | Suzhou | Vaginal secretion | MZ172596 | D |
| 298 | *Candida albicans* | Suzhou | Vaginal secretion | MZ172607 | D |
| 250 | *Candida albicans* | Suzhou | Vaginal secretion | MZ172579 | E |
| 258 | *Candida albicans* | Suzhou | Vaginal secretion | MZ172585 | E |
| 295 | *Candida albicans* | Suzhou | Vaginal secretion | MZ172604 | E |
| 300 | *Candida albicans* | Suzhou | Vaginal secretion | MZ172610 | E |
| 320 | *Candida albicans* | Suzhou | Vaginal secretion | MZ172626 | E |
| 69 | *Candida albicans* | Suzhou | Vaginal secretion | MZ172675 | E |
| 141 | *Candida albicans* | Suzhou | Vaginal secretion | MZ172489 | F |
| 144 | *Candida albicans* | Suzhou | Vaginal secretion | MZ172492 | F |
| 314 | *Candida albicans* | Suzhou | Vaginal secretion | MZ172621 | F |
| 389 | *Candida albicans* | Suzhou | Vaginal secretion | MZ172645 | F |
| 90 | *Candida albicans* | Suzhou | Vaginal secretion | MZ172694 | F |
| 103 | *Candida albicans* | Suzhou | Vaginal secretion | MZ172464 | Other genotypes |
| 107 | *Candida albicans* | Suzhou | Vaginal secretion | MZ172468 | Other genotypes |
| 12 | *Candida albicans* | Suzhou | Vaginal secretion | MZ172481 | Other genotypes |
| 135 | *Candida albicans* | Suzhou | Vaginal secretion | MZ172483 | Other genotypes |
| 137 | *Candida albicans* | Suzhou | Vaginal secretion | MZ172485 | Other genotypes |
| 147 | *Candida albicans* | Suzhou | Vaginal secretion | MZ172494 | Other genotypes |
| 149 | *Candida albicans* | Suzhou | Vaginal secretion | MZ172496 | Other genotypes |
| 152 | *Candida albicans* | Suzhou | Vaginal secretion | MZ172498 | Other genotypes |
| 181 | *Candida albicans* | Suzhou | Vaginal secretion | MZ172520 | Other genotypes |
| 186 | *Candida albicans* | Suzhou | Vaginal secretion | MZ172524 | Other genotypes |
| 19 | *Candida albicans* | Suzhou | Vaginal secretion | MZ172536 | Other genotypes |
| 200 | *Candida albicans* | Suzhou | Vaginal secretion | MZ172537 | Other genotypes |
| 202 | *Candida albicans* | Suzhou | Vaginal secretion | MZ172539 | Other genotypes |
| 203 | *Candida albicans* | Suzhou | Vaginal secretion | MZ226435 | Other genotypes |
| 207 | *Candida albicans* | Suzhou | Vaginal secretion | MZ172543 | Other genotypes |
| 210 | *Candida albicans* | Suzhou | Vaginal secretion | MZ172547 | Other genotypes |
| 214 | *Candida albicans* | Suzhou | Vaginal secretion | MZ172549 | Other genotypes |
| 225 | *Candida albicans* | Suzhou | Vaginal secretion | MZ172556 | Other genotypes |
| 228 | *Candida albicans* | Suzhou | Vaginal secretion | MZ172559 | Other genotypes |
| 233 | *Candida albicans* | Suzhou | Vaginal secretion | MZ172562 | Other genotypes |
| 239 | *Candida albicans* | Suzhou | Vaginal secretion | MZ172568 | Other genotypes |
| 253 | *Candida albicans* | Suzhou | Vaginal secretion | MZ226436 | Other genotypes |
| 257 | *Candida albicans* | Suzhou | Vaginal secretion | MZ172584 | Other genotypes |
| 286 | *Candida albicans* | Suzhou | Vaginal secretion | MZ172595 | Other genotypes |
| 294 | *Candida albicans* | Suzhou | Vaginal secretion | MZ172603 | Other genotypes |
| 296 | *Candida albicans* | Suzhou | Vaginal secretion | MZ172605 | Other genotypes |
| 299 | *Candida albicans* | Suzhou | Vaginal secretion | MZ172653 | Other genotypes |
| 305 | *Candida albicans* | Suzhou | Vaginal secretion | MZ172614 | Other genotypes |
| 312 | *Candida albicans* | Suzhou | Vaginal secretion | MZ172619 | Other genotypes |
| 317 | *Candida albicans* | Suzhou | Vaginal secretion | MZ172624 | Other genotypes |
| 321 | *Candida albicans* | Suzhou | Vaginal secretion | MZ172627 | Other genotypes |
| 426 | *Candida albicans* | Suzhou | Vaginal secretion | MZ172663 | Other genotypes |
| 61 | *Candida albicans* | Suzhou | Vaginal secretion | MZ172671 | Other genotypes |
| 63 | *Candida albicans* | Suzhou | Vaginal secretion | MZ172672 | Other genotypes |
| 70 | *Candida albicans* | Suzhou | Vaginal secretion | MZ172677 | Other genotypes |
| 73 | *Candida albicans* | Suzhou | Vaginal secretion | MZ172680 | Other genotypes |
| 78 | *Candida albicans* | Suzhou | Vaginal secretion | MZ172685 | Other genotypes |
| 83 | *Candida albicans* | Suzhou | Vaginal secretion | MZ172689 | Other genotypes |
| 121 | *Candida albicans* | Suzhou | Vaginal secretion | MZ172475 | Unique genotype |
| 125 | *Candida albicans* | Suzhou | Vaginal secretion | MZ172478 | Unique genotype |
| 136 | *Candida albicans* | Suzhou | Vaginal secretion | MZ172484 | Unique genotype |
| 142 | *Candida albicans* | Suzhou | Vaginal secretion | MZ172490 | Unique genotype |
| 143 | *Candida albicans* | Suzhou | Vaginal secretion | MZ172491 | Unique genotype |
| 15 | *Candida albicans* | Suzhou | Vaginal secretion | MZ172502 | Unique genotype |
| 156 | *Candida albicans* | Suzhou | Vaginal secretion | MZ172500 | Unique genotype |
| 158 | *Candida albicans* | Suzhou | Vaginal secretion | MZ172501 | Unique genotype |
| 160 | *Candida albicans* | Suzhou | Vaginal secretion | MZ172503 | Unique genotype |
| 166 | *Candida albicans* | Suzhou | Vaginal secretion | MZ172507 | Unique genotype |
| 169 | *Candida albicans* | Suzhou | Vaginal secretion | MZ172509 | Unique genotype |
| 174 | *Candida albicans* | Suzhou | Vaginal secretion | MZ172514 | Unique genotype |
| 178 | *Candida albicans* | Suzhou | Vaginal secretion | MZ172517 | Unique genotype |
| 180 | *Candida albicans* | Suzhou | Vaginal secretion | MZ172519 | Unique genotype |
| 192 | *Candida albicans* | Suzhou | Vaginal secretion | MZ172529 | Unique genotype |
| 198 | *Candida albicans* | Suzhou | Vaginal secretion | MZ172534 | Unique genotype |
| 199 | *Candida albicans* | Suzhou | Vaginal secretion | MZ172535 | Unique genotype |
| 201 | *Candida albicans* | Suzhou | Vaginal secretion | MZ172538 | Unique genotype |
| 215 | *Candida albicans* | Suzhou | Vaginal secretion | MZ172550 | Unique genotype |
| 235 | *Candida albicans* | Suzhou | Vaginal secretion | MZ172564 | Unique genotype |
| 238 | *Candida albicans* | Suzhou | Vaginal secretion | MZ172567 | Unique genotype |
| 242 | *Candida albicans* | Suzhou | Vaginal secretion | MZ172572 | Unique genotype |
| 252 | *Candida albicans* | Suzhou | Vaginal secretion | MZ172581 | Unique genotype |
| 259 | *Candida albicans* | Suzhou | Vaginal secretion | MZ172586 | Unique genotype |
| 26 | *Candida albicans* | Suzhou | Vaginal secretion | MZ172588 | Unique genotype |
| 277 | *Candida albicans* | Suzhou | Vaginal secretion | MZ172589 | Unique genotype |
| 280 | *Candida albicans* | Suzhou | Vaginal secretion | MZ226437 | Unique genotype |
| 288 | *Candida albicans* | Suzhou | Vaginal secretion | MZ172597 | Unique genotype |
| 291 | *Candida albicans* | Suzhou | Vaginal secretion | MZ172600 | Unique genotype |
| 310 | *Candida albicans* | Suzhou | Vaginal secretion | MZ172617 | Unique genotype |
| 315 | *Candida albicans* | Suzhou | Vaginal secretion | MZ172622 | Unique genotype |
| 316 | *Candida albicans* | Suzhou | Vaginal secretion | MZ172623 | Unique genotype |
| 322 | *Candida albicans* | Suzhou | Vaginal secretion | MZ172628 | Unique genotype |
| 325 | *Candida albicans* | Suzhou | Vaginal secretion | MZ172630 | Unique genotype |
| 330 | *Candida albicans* | Suzhou | Vaginal secretion | MZ172635 | Unique genotype |
| 384 | *Candida albicans* | Suzhou | Vaginal secretion | MZ172641 | Unique genotype |
| 392 | *Candida albicans* | Suzhou | Vaginal secretion | MZ172647 | Unique genotype |
| 397 | *Candida albicans* | Suzhou | Vaginal secretion | MZ172649 | Unique genotype |
| 407 | *Candida albicans* | Suzhou | Vaginal secretion | MZ172657 | Unique genotype |
| 414 | *Candida albicans* | Suzhou | Vaginal secretion | MZ172660 | Unique genotype |
| 46 | *Candida albicans* | Suzhou | Vaginal secretion | MZ172665 | Unique genotype |
| 64 | *Candida albicans* | Suzhou | Vaginal secretion | MZ172673 | Unique genotype |
| 67 | *Candida albicans* | Suzhou | Vaginal secretion | MZ172674 | Unique genotype |
| 71 | *Candida albicans* | Suzhou | Vaginal secretion | MZ172678 | Unique genotype |
| 216 | *Candida albicans* | Suzhou | Vaginal secretion | MZ172551 | Unique genotype |
| 227 | *Candida albicans* | Suzhou | Vaginal secretion | MZ172558 | Unique genotype |
| 106 | *Candida albicans* | Suzhou | Vaginal secretion | MZ172467 | Unique genotype |
| 109 | *Candida albicans* | Suzhou | Vaginal secretion | MZ172469 | Unique genotype |
| 11 | *Candida albicans* | Suzhou | Vaginal secretion | MZ172474 | Unique genotype |
| 112 | *Candida albicans* | Suzhou | Vaginal secretion | MZ172470 | Unique genotype |
| 113 | *Candida albicans* | Suzhou | Vaginal secretion | MZ172471 | Unique genotype |
| 115 | *Candida albicans* | Suzhou | Vaginal secretion | MZ172472 | Unique genotype |
| 123 | *Candida albicans* | Suzhou | Vaginal secretion | MZ172477 | Unique genotype |
| 126 | *Candida albicans* | Suzhou | Vaginal secretion | MZ172479 | Unique genotype |
| 130 | *Candida albicans* | Suzhou | Vaginal secretion | MZ172482 | Unique genotype |
| 150 | *Candida albicans* | Suzhou | Vaginal secretion | MZ172497 | Unique genotype |
| 155 | *Candida albicans* | Suzhou | Vaginal secretion | MZ172499 | Unique genotype |
| 161 | *Candida albicans* | Suzhou | Vaginal secretion | MZ172504 | Unique genotype |
| 168 | *Candida albicans* | Suzhou | Vaginal secretion | MZ172508 | Unique genotype |
| 170 | *Candida albicans* | Suzhou | Vaginal secretion | MZ172510 | Unique genotype |
| 171 | *Candida albicans* | Suzhou | Vaginal secretion | MZ172511 | Unique genotype |
| 173 | *Candida albicans* | Suzhou | Vaginal secretion | MZ172513 | Unique genotype |
| 175 | *Candida albicans* | Suzhou | Vaginal secretion | MZ172515 | Unique genotype |
| 179 | *Candida albicans* | Suzhou | Vaginal secretion | MZ172518 | Unique genotype |
| 184 | *Candida albicans* | Suzhou | Vaginal secretion | MZ172522 | Unique genotype |
| 191 | *Candida albicans* | Suzhou | Vaginal secretion | MZ172528 | Unique genotype |
| 194 | *Candida albicans* | Suzhou | Vaginal secretion | MZ172531 | Unique genotype |
| 204 | *Candida albicans* | Suzhou | Vaginal secretion | MZ172540 | Unique genotype |
| 223 | *Candida albicans* | Suzhou | Vaginal secretion | MZ172554 | Unique genotype |
| 226 | *Candida albicans* | Suzhou | Vaginal secretion | MZ172557 | Unique genotype |
| 241 | *Candida albicans* | Suzhou | Vaginal secretion | MZ172571 | Unique genotype |
| 243 | *Candida albicans* | Suzhou | Vaginal secretion | MZ172573 | Unique genotype |
| 244 | *Candida albicans* | Suzhou | Vaginal secretion | MZ172574 | Unique genotype |
| 245 | *Candida albicans* | Suzhou | Vaginal secretion | MZ172575 | Unique genotype |
| 247 | *Candida albicans* | Suzhou | Vaginal secretion | MZ172576 | Unique genotype |
| 251 | *Candida albicans* | Suzhou | Vaginal secretion | MZ172580 | Unique genotype |
| 254 | *Candida albicans* | Suzhou | Vaginal secretion | MZ172582 | Unique genotype |
| 256 | *Candida albicans* | Suzhou | Vaginal secretion | MZ172583 | Unique genotype |
| 260 | *Candida albicans* | Suzhou | Vaginal secretion | MZ172587 | Unique genotype |
| 278 | *Candida albicans* | Suzhou | Vaginal secretion | MZ172590 | Unique genotype |
| 283 | *Candida albicans* | Suzhou | Vaginal secretion | MZ172592 | Unique genotype |
| 290 | *Candida albicans* | Suzhou | Vaginal secretion | MZ172599 | Unique genotype |
| 292 | *Candida albicans* | Suzhou | Vaginal secretion | MZ172601 | Unique genotype |
| 293 | *Candida albicans* | Suzhou | Vaginal secretion | MZ172602 | Unique genotype |
| 3 | *Candida albicans* | Suzhou | Vaginal secretion | MZ172609 | Unique genotype |
| 304 | *Candida albicans* | Suzhou | Vaginal secretion | MZ172613 | Unique genotype |
| 308 | *Candida albicans* | Suzhou | Vaginal secretion | MZ172616 | Unique genotype |
| 311 | *Candida albicans* | Suzhou | Vaginal secretion | MZ172618 | Unique genotype |
| 313 | *Candida albicans* | Suzhou | Vaginal secretion | MZ172620 | Unique genotype |
| 332 | *Candida albicans* | Suzhou | Vaginal secretion | MZ172637 | Unique genotype |
| 333 | *Candida albicans* | Suzhou | Vaginal secretion | MZ172638 | Unique genotype |
| 36 | *Candida albicans* | Suzhou | Vaginal secretion | MZ172666 | Unique genotype |
| 386 | *Candida albicans* | Suzhou | Vaginal secretion | MZ172643 | Unique genotype |
| 398 | *Candida albicans* | Suzhou | Vaginal secretion | MZ172650 | Unique genotype |
| 406 | *Candida albicans* | Suzhou | Vaginal secretion | MZ172656 | Unique genotype |
| 415 | *Candida albicans* | Suzhou | Vaginal secretion | MZ172661 | Unique genotype |
| 49 | *Candida albicans* | Suzhou | Vaginal secretion | MZ172667 | Unique genotype |
| 6 | *Candida albicans* | Suzhou | Vaginal secretion | MZ172676 | Unique genotype |
| 72 | *Candida albicans* | Suzhou | Vaginal secretion | MZ172679 | Unique genotype |
| 76 | *Candida albicans* | Suzhou | Vaginal secretion | MZ172683 | Unique genotype |
| 77 | *Candida albicans* | Suzhou | Vaginal secretion | MZ172684 | Unique genotype |
| 80 | *Candida albicans* | Suzhou | Vaginal secretion | MZ172687 | Unique genotype |
| 86 | *Candida albicans* | Suzhou | Vaginal secretion | MZ172691 | Unique genotype |
| 87 | *Candida albicans* | Suzhou | Vaginal secretion | MZ172692 | Unique genotype |
| 89 | *Candida albicans* | Suzhou | Vaginal secretion | MZ172693 | Unique genotype |
| 91 | *Candida albicans* | Suzhou | Vaginal secretion | MZ172695 | Unique genotype |
| 95 | *Candida albicans* | Suzhou | Vaginal secretion | MZ172698 | Unique genotype |
| 98 | *Candida albicans* | Suzhou | Vaginal secretion | MZ172700 | Unique genotype |

Note: other genotype: means the genotype consisting of less than 5 isolates; unique genotype: the genotype consisting of 1 isolate, Nr. : number.
