# Supplementary material for: Genotyping and Drug Resistance Profile of Clinical Isolates of Candida albicans from Vulvovaginal Candidiasis in the Eastern China
Source: Mycopathologia. 2022 Jan 24;187(2-3):217–24. doi: 10.1007/s11046-022-00616-x (PMC9124162; doi:10.1007/s11046-022-00616-x)
Supplement: Supplementary file 2 — Supplementary file2 (DOCX 17 kb) [file 11046_2022_616_MOESM2_ESM.docx]

| **Supplementary table 2** Drug concentration range、time of MIC reading、interpretive breakpoints for 9 antifungal agents tested in this study | | | | | | |  |
| --- | --- | --- | --- | --- | --- | --- | --- |
|  |  |  |  |  |  |  |  |
| Drugs | Drug concentration range (mg/L) | Time of MIC reading | Clinical breakpoints (mg/L) | | | | ECV (mg/L) |
|  |  |  | S≦ | I | SDD | R≧ | WT≦ |
| Anidulafungin | 0.016-8 | 24h | 0.25 | 0.5 | NA | 1 | 0.12 |
| Caspofungin | 0.016-8 | 24h | 0.25 | 0.5 | NA | 1 | NA |
| Micafungin | 0.016-8 | 24h | 0.25 | 0.5 | NA | 1 | 0.03 |
| Voriconazole | 0.0313-16 | 48h | 0.125 | 0.25-0.5 | NA | 1 | 0.03 |
| Fluconazole | 0.0313-16 | 24h | 2 | NA | 4 | 8 | 0.5 |
| Posaconazole | 0.0313-16 | 48h | NA | NA | NA | NA | 0.06 |
| Amphotericine B | 0.0313-16 | 24h | NA | NA | NA | NA | 2 |
| 5-flucytosine | 0.125-64 | 48h | NA^a^ | NA | NA | NA | NA |
| Itraconazole | 0.0313-16 | 48h | NA | NA | NA | NA | NA |

Abbreviations: S: susceptible; I: intermediate; SDD: susceptible-dose dependent; R: resistant; ECV: epidemiological cutoff values; WT: wildtype; a: not applicable
