# Supplementary material for: Genotyping and Drug Resistance Profile of Clinical Isolates of Candida albicans from Vulvovaginal Candidiasis in the Eastern China
Source: Mycopathologia. 2022 Jan 24;187(2-3):217–24. doi: 10.1007/s11046-022-00616-x (PMC9124162; doi:10.1007/s11046-022-00616-x)
Supplement: Supplementary file 3 — Supplementary file3 (DOCX 53 kb) [file 11046_2022_616_MOESM3_ESM.docx]

Stable 3 Microsatellite genotyping data on different alleles and allelic combination in the 244 *C. albicans* isolates tested in this study

| **Sample Nr.** | **CEF3_Peak1** | **CEF3_Peak 2** | **CAIII_Peak1** | **CAIII_Peak2** | **LOC4_Peak1** | **LOC4_Peak 2** | **Allelic combination** | **Clade** |
| --- | --- | --- | --- | --- | --- | --- | --- | --- |
| sample_100 | 124 | 134 | 112 | 113 | 119 | 122 | 1 | clade1 |
| sample_122 | 124 | 134 | 112 | 113 | 119 | 122 | 1 | clade1 |
| sample_129 | 124 | 134 | 112 | 113 | 119 | 122 | 1 | clade1 |
| sample_139 | 124 | 134 | 112 | 113 | 119 | 122 | 1 | clade1 |
| sample_140 | 124 | 134 | 112 | 113 | 119 | 122 | 1 | clade1 |
| sample_162 | 124 | 134 | 112 | 113 | 119 | 122 | 1 | clade1 |
| sample_190 | 124 | 134 | 112 | 113 | 119 | 122 | 1 | clade1 |
| sample_2 | 124 | 134 | 112 | 113 | 119 | 122 | 1 | clade1 |
| sample_20 | 124 | 134 | 112 | 113 | 119 | 122 | 1 | clade1 |
| sample_206 | 124 | 134 | 112 | 113 | 119 | 122 | 1 | clade1 |
| sample_212 | 124 | 134 | 112 | 113 | 119 | 122 | 1 | clade1 |
| sample_217 | 124 | 134 | 112 | 113 | 119 | 122 | 1 | clade1 |
| sample_229 | 124 | 134 | 112 | 113 | 119 | 122 | 1 | clade1 |
| sample_231 | 124 | 134 | 112 | 113 | 119 | 122 | 1 | clade1 |
| sample_248 | 124 | 134 | 112 | 113 | 119 | 122 | 1 | clade1 |
| sample_284 | 124 | 134 | 112 | 113 | 119 | 122 | 1 | clade1 |
| sample_289 | 124 | 134 | 112 | 113 | 119 | 122 | 1 | clade1 |
| sample_323 | 124 | 134 | 112 | 113 | 119 | 122 | 1 | clade1 |
| sample_327 | 124 | 134 | 112 | 113 | 119 | 122 | 1 | clade1 |
| sample_37 | 124 | 134 | 112 | 113 | 119 | 122 | 1 | clade1 |
| sample_385 | 124 | 134 | 112 | 113 | 119 | 122 | 1 | clade1 |
| sample_39 | 124 | 134 | 112 | 113 | 119 | 122 | 1 | clade1 |
| sample_391 | 124 | 134 | 112 | 113 | 119 | 122 | 1 | clade1 |
| sample_396 | 124 | 134 | 112 | 113 | 119 | 122 | 1 | clade1 |
| sample_404 | 124 | 134 | 112 | 113 | 119 | 122 | 1 | clade1 |
| sample_45 | 124 | 134 | 112 | 113 | 119 | 122 | 1 | clade1 |
| sample_58 | 124 | 134 | 112 | 113 | 119 | 122 | 1 | clade1 |
| sample_60 | 124 | 134 | 112 | 113 | 119 | 122 | 1 | clade1 |
| sample_75 | 124 | 134 | 112 | 113 | 119 | 122 | 1 | clade1 |
| sample_81 | 124 | 134 | 112 | 113 | 119 | 122 | 1 | clade1 |
| sample_9 | 124 | 134 | 112 | 113 | 119 | 122 | 1 | clade1 |
| sample_92 | 124 | 134 | 112 | 113 | 119 | 122 | 1 | clade1 |
| sample_96 | 124 | 134 | 112 | 113 | 119 | 122 | 1 | clade1 |
| sample_99 | 124 | 134 | 112 | 113 | 119 | 122 | 1 | clade1 |
| sample_104 | 124 | 134 | 98 | 112 | 119 | 122 | 2 | clade2 |
| sample_105 | 124 | 134 | 98 | 112 | 119 | 122 | 2 | clade2 |
| sample_116 | 124 | 134 | 98 | 112 | 119 | 122 | 2 | clade2 |
| sample_138 | 124 | 134 | 98 | 112 | 119 | 122 | 2 | clade2 |
| sample_148 | 124 | 134 | 98 | 112 | 119 | 122 | 2 | clade2 |
| sample_208 | 124 | 134 | 98 | 112 | 119 | 122 | 2 | clade2 |
| sample_220 | 124 | 134 | 98 | 112 | 119 | 122 | 2 | clade2 |
| sample_297 | 124 | 134 | 98 | 112 | 119 | 122 | 2 | clade2 |
| sample_303 | 124 | 134 | 98 | 112 | 119 | 122 | 2 | clade2 |
| sample_306 | 124 | 134 | 98 | 112 | 119 | 122 | 2 | clade2 |
| sample_319 | 124 | 134 | 98 | 112 | 119 | 122 | 2 | clade2 |
| sample_326 | 124 | 134 | 98 | 112 | 119 | 122 | 2 | clade2 |
| sample_328 | 124 | 134 | 98 | 112 | 119 | 122 | 2 | clade2 |
| sample_329 | 124 | 134 | 98 | 112 | 119 | 122 | 2 | clade2 |
| sample_331 | 124 | 134 | 98 | 112 | 119 | 122 | 2 | clade2 |
| sample_334 | 124 | 134 | 98 | 112 | 119 | 122 | 2 | clade2 |
| sample_388 | 124 | 134 | 98 | 112 | 119 | 122 | 2 | clade2 |
| sample_400 | 124 | 134 | 98 | 112 | 119 | 122 | 2 | clade2 |
| sample_405 | 124 | 134 | 98 | 112 | 119 | 122 | 2 | clade2 |
| sample_411 | 124 | 134 | 98 | 112 | 119 | 122 | 2 | clade2 |
| sample_412 | 124 | 134 | 98 | 112 | 119 | 122 | 2 | clade2 |
| sample_416 | 124 | 134 | 98 | 112 | 119 | 122 | 2 | clade2 |
| sample_57 | 124 | 134 | 98 | 112 | 119 | 122 | 2 | clade2 |
| sample_74 | 124 | 134 | 98 | 112 | 119 | 122 | 2 | clade2 |
| sample_94 | 124 | 134 | 98 | 112 | 119 | 122 | 2 | clade2 |
| sample_101 | 124 | 134 | 112 | 112 | 119 | 122 | 3 | clade1 |
| sample_172 | 124 | 134 | 112 | 112 | 119 | 122 | 3 | clade1 |
| sample_182 | 124 | 134 | 112 | 112 | 119 | 122 | 3 | clade1 |
| sample_185 | 124 | 134 | 112 | 112 | 119 | 122 | 3 | clade1 |
| sample_188 | 124 | 134 | 112 | 112 | 119 | 122 | 3 | clade1 |
| sample_189 | 124 | 134 | 112 | 112 | 119 | 122 | 3 | clade1 |
| sample_197 | 124 | 134 | 112 | 112 | 119 | 122 | 3 | clade1 |
| sample_205 | 124 | 134 | 112 | 112 | 119 | 122 | 3 | clade1 |
| sample_209 | 124 | 134 | 112 | 112 | 119 | 122 | 3 | clade1 |
| sample_234 | 124 | 134 | 112 | 112 | 119 | 122 | 3 | clade1 |
| sample_236 | 124 | 134 | 112 | 112 | 119 | 122 | 3 | clade1 |
| sample_237 | 124 | 134 | 112 | 112 | 119 | 122 | 3 | clade1 |
| sample_240 | 124 | 134 | 112 | 112 | 119 | 122 | 3 | clade1 |
| sample_249 | 124 | 134 | 112 | 112 | 119 | 122 | 3 | clade1 |
| sample_285 | 124 | 134 | 112 | 112 | 119 | 122 | 3 | clade1 |
| sample_301 | 124 | 134 | 112 | 112 | 119 | 122 | 3 | clade1 |
| sample_79 | 124 | 134 | 112 | 112 | 119 | 122 | 3 | clade1 |
| sample_85 | 124 | 134 | 112 | 112 | 119 | 122 | 3 | clade1 |
| sample_146 | 124 | 134 | 99 | 113 | 119 | 122 | 4 | clade2 |
| sample_163 | 124 | 134 | 99 | 113 | 119 | 122 | 4 | clade2 |
| sample_176 | 124 | 134 | 99 | 113 | 119 | 122 | 4 | clade2 |
| sample_193 | 124 | 134 | 99 | 113 | 119 | 122 | 4 | clade2 |
| sample_196 | 124 | 134 | 99 | 113 | 119 | 122 | 4 | clade2 |
| sample_22 | 124 | 134 | 99 | 113 | 119 | 122 | 4 | clade2 |
| sample_224 | 124 | 134 | 99 | 113 | 119 | 122 | 4 | clade2 |
| sample_281 | 124 | 134 | 99 | 113 | 119 | 122 | 4 | clade2 |
| sample_287 | 124 | 134 | 99 | 113 | 119 | 122 | 4 | clade2 |
| sample_298 | 124 | 134 | 99 | 113 | 119 | 122 | 4 | clade2 |
| sample_250 | 127 | 127 | 110 | 110 | 122 | 134 | 5 | clade4 |
| sample_258 | 127 | 127 | 110 | 110 | 122 | 134 | 5 | clade4 |
| sample_295 | 127 | 127 | 110 | 110 | 122 | 134 | 5 | clade4 |
| sample_300 | 127 | 127 | 110 | 110 | 122 | 134 | 5 | clade4 |
| sample_320 | 127 | 127 | 110 | 110 | 122 | 134 | 5 | clade4 |
| sample_69 | 127 | 127 | 110 | 110 | 122 | 134 | 5 | clade4 |
| sample_141 | 127 | 127 | 110 | 111 | 122 | 134 | 6 | clade4 |
| sample_144 | 127 | 127 | 110 | 111 | 122 | 134 | 6 | clade4 |
| sample_314 | 127 | 127 | 110 | 111 | 122 | 134 | 6 | clade4 |
| sample_389 | 127 | 127 | 110 | 111 | 122 | 134 | 6 | clade4 |
| sample_90 | 127 | 127 | 110 | 111 | 122 | 134 | 6 | clade4 |
| sample_147 | 124 | 134 | 113 | 113 | 119 | 122 | 7 | clade1 |
| sample_19 | 124 | 134 | 113 | 113 | 119 | 122 | 7 | clade1 |
| sample_286 | 124 | 134 | 113 | 113 | 119 | 122 | 7 | clade1 |
| sample_294 | 124 | 134 | 113 | 113 | 119 | 122 | 7 | clade1 |
| sample_207 | 124 | 134 | 110 | 112 | 119 | 122 | 8 | clade1 |
| sample_253 | 124 | 134 | 110 | 112 | 119 | 122 | 8 | clade1 |
| sample_257 | 124 | 134 | 110 | 112 | 119 | 122 | 8 | clade1 |
| sample_107 | 134 | 134 | 112 | 113 | 119 | 122 | 9 | clade1 |
| sample_233 | 134 | 134 | 112 | 113 | 119 | 122 | 9 | clade1 |
| sample_321 | 134 | 134 | 112 | 113 | 119 | 122 | 9 | clade1 |
| sample_137 | 124 | 134 | 98 | 113 | 119 | 122 | 10 | clade2 |
| sample_210 | 124 | 134 | 98 | 113 | 119 | 122 | 10 | clade2 |
| sample_225 | 124 | 134 | 98 | 113 | 119 | 122 | 10 | clade2 |
| sample_200 | 124 | 127 | 99 | 113 | 119 | 122 | 11 | clade2 |
| sample_73 | 124 | 127 | 99 | 113 | 119 | 122 | 11 | clade2 |
| sample_78 | 124 | 127 | 99 | 113 | 119 | 122 | 11 | clade2 |
| sample_103 | 124 | 134 | 96 | 99 | 119 | 122 | 12 | clade5 |
| sample_186 | 124 | 134 | 96 | 99 | 119 | 122 | 12 | clade5 |
| sample_202 | 124 | 134 | 96 | 99 | 119 | 122 | 12 | clade5 |
| sample_135 | 128 | 132 | 95 | 104 | 122 | 131 | 13 | clade5 |
| sample_296 | 128 | 132 | 95 | 104 | 122 | 131 | 13 | clade5 |
| sample_63 | 128 | 132 | 95 | 104 | 122 | 131 | 13 | clade5 |
| sample_203 | 124 | 134 | 111 | 112 | 119 | 122 | 14 | clade1 |
| sample_228 | 124 | 134 | 111 | 112 | 119 | 122 | 14 | clade1 |
| sample_152 | 124 | 134 | 112 | 113 | 119 | 123 | 15 | clade1 |
| sample_83 | 124 | 134 | 112 | 113 | 119 | 123 | 15 | clade1 |
| sample_214 | 124 | 134 | 99 | 112 | 119 | 122 | 16 | clade2 |
| sample_239 | 124 | 134 | 99 | 112 | 119 | 122 | 16 | clade2 |
| sample_12 | 124 | 134 | 96 | 113 | 119 | 122 | 17 | clade2 |
| sample_70 | 124 | 134 | 96 | 113 | 119 | 122 | 17 | clade2 |
| sample_299 | 124 | 127 | 98 | 112 | 119 | 122 | 18 | clade2 |
| sample_312 | 124 | 127 | 98 | 112 | 119 | 122 | 18 | clade2 |
| sample_181 | 124 | 128 | 96 | 113 | 119 | 122 | 19 | clade2 |
| sample_426 | 124 | 128 | 96 | 113 | 119 | 122 | 19 | clade2 |
| sample_149 | 135 | 144 | 98 | 106 | 119 | 131 | 20 | clade6 |
| sample_305 | 135 | 144 | 98 | 106 | 119 | 131 | 20 | clade6 |
| sample_317 | 142 | 142 | 95 | 95 | 119 | 122 | 21 | clade6 |
| sample_61 | 142 | 142 | 95 | 95 | 119 | 122 | 21 | clade6 |
| sample_87 | 124 | 134 | 112 | 112 | 119 | 123 | 22 | clade1 |
| sample_77 | 124 | 134 | 109 | 112 | 119 | 122 | 23 | clade1 |
| sample_143 | 124 | 134 | 112 | 112 | 122 | 122 | 24 | clade1 |
| sample_158 | 124 | 134 | 113 | 113 | 122 | 122 | 25 | clade1 |
| sample_280 | 123 | 126 | 112 | 113 | 119 | 122 | 26 | clade1 |
| sample_325 | 124 | 127 | 112 | 113 | 119 | 122 | 27 | clade1 |
| sample_311 | 122 | 126 | 112 | 112 | 119 | 122 | 28 | clade1 |
| sample_106 | 124 | 124 | 112 | 112 | 119 | 122 | 29 | clade1 |
| sample_126 | 124 | 134 | 98 | 112 | 119 | 123 | 30 | clade2 |
| sample_290 | 124 | 134 | 99 | 110 | 119 | 122 | 31 | clade2 |
| sample_223 | 124 | 134 | 95 | 113 | 119 | 122 | 32 | clade2 |
| sample_72 | 124 | 134 | 95 | 112 | 119 | 122 | 33 | clade2 |
| sample_113 | 124 | 137 | 98 | 112 | 119 | 122 | 34 | clade2 |
| sample_243 | 124 | 134 | 99 | 113 | 119 | 119 | 35 | clade2 |
| sample_244 | 124 | 128 | 99 | 113 | 119 | 122 | 36 | clade2 |
| sample_278 | 124 | 127 | 99 | 110 | 119 | 122 | 37 | clade2 |
| sample_204 | 124 | 130 | 99 | 113 | 119 | 122 | 38 | clade2 |
| sample_6 | 124 | 127 | 96 | 113 | 119 | 122 | 39 | clade2 |
| sample_3 | 124 | 127 | 96 | 112 | 119 | 122 | 40 | clade2 |
| sample_245 | 127 | 135 | 96 | 113 | 116 | 119 | 41 | clade2 |
| sample_259 | 113 | 127 | 99 | 108 | 119 | 122 | 42 | clade3 |
| sample_315 | 113 | 124 | 99 | 113 | 119 | 122 | 43 | clade3 |
| sample_333 | 120 | 124 | 96 | 113 | 119 | 122 | 44 | clade3 |
| sample_175 | 117 | 124 | 110 | 113 | 119 | 122 | 45 | clade3 |
| sample_247 | 124 | 129 | 97 | 112 | 127 | 127 | 46 | clade5 |
| sample_173 | 127 | 127 | 109 | 110 | 122 | 134 | 47 | clade4 |
| sample_256 | 128 | 128 | 107 | 110 | 122 | 134 | 48 | clade4 |
| sample_283 | 124 | 128 | 111 | 113 | 122 | 134 | 49 | clade4 |
| sample_292 | 127 | 127 | 107 | 110 | 122 | 128 | 50 | clade4 |
| sample_241 | 126 | 134 | 96 | 99 | 119 | 122 | 51 | clade5 |
| sample_293 | 124 | 134 | 95 | 98 | 119 | 122 | 52 | clade5 |
| sample_171 | 124 | 134 | 98 | 98 | 119 | 122 | 53 | clade5 |
| sample_398 | 124 | 134 | 95 | 98 | 121 | 124 | 54 | clade5 |
| sample_174 | 126 | 130 | 96 | 99 | 119 | 122 | 55 | clade5 |
| sample_277 | 124 | 129 | 96 | 99 | 119 | 122 | 56 | clade5 |
| sample_179 | 124 | 130 | 96 | 99 | 122 | 122 | 57 | clade5 |
| sample_322 | 124 | 127 | 95 | 98 | 119 | 122 | 58 | clade5 |
| sample_384 | 124 | 131 | 95 | 98 | 119 | 122 | 59 | clade5 |
| sample_313 | 124 | 130 | 95 | 104 | 119 | 122 | 60 | clade5 |
| sample_254 | 127 | 127 | 96 | 99 | 122 | 122 | 61 | clade5 |
| sample_304 | 128 | 132 | 95 | 98 | 116 | 119 | 62 | clade5 |
| sample_180 | 132 | 136 | 98 | 99 | 119 | 122 | 63 | clade5 |
| sample_414 | 134 | 134 | 99 | 99 | 119 | 122 | 64 | clade5 |
| sample_161 | 134 | 134 | 94 | 99 | 118 | 123 | 65 | clade5 |
| sample_226 | 130 | 135 | 98 | 98 | 116 | 122 | 66 | clade5 |
| sample_215 | 120 | 124 | 96 | 99 | 119 | 122 | 67 | clade5 |
| sample_330 | 119 | 124 | 95 | 99 | 119 | 122 | 68 | clade5 |
| sample_406 | 113 | 124 | 95 | 98 | 119 | 122 | 69 | clade5 |
| sample_191 | 118 | 124 | 98 | 99 | 116 | 119 | 70 | clade5 |
| sample_251 | 113 | 134 | 96 | 99 | 119 | 122 | 71 | clade5 |
| sample_216 | 128 | 132 | 96 | 104 | 122 | 131 | 72 | clade5 |
| sample_227 | 128 | 132 | 95 | 103 | 122 | 131 | 73 | clade5 |
| sample_15 | 129 | 132 | 95 | 104 | 122 | 131 | 74 | clade5 |
| sample_178 | 129 | 132 | 96 | 104 | 122 | 131 | 75 | clade5 |
| sample_136 | 128 | 132 | 104 | 104 | 122 | 131 | 76 | clade5 |
| sample_160 | 128 | 132 | 104 | 105 | 122 | 131 | 77 | clade5 |
| sample_36 | 130 | 130 | 98 | 106 | 119 | 131 | 78 | clade5 |
| sample_194 | 128 | 131 | 96 | 103 | 131 | 131 | 79 | clade5 |
| sample_142 | 124 | 127 | 95 | 98 | 122 | 134 | 80 | clade5 |
| sample_397 | 124 | 128 | 95 | 98 | 119 | 131 | 81 | clade5 |
| sample_184 | 128 | 128 | 96 | 96 | 122 | 131 | 82 | clade5 |
| sample_198 | 128 | 140 | 99 | 107 | 122 | 122 | 83 | clade6 |
| sample_392 | 128 | 140 | 98 | 106 | 122 | 122 | 84 | clade6 |
| sample_125 | 128 | 140 | 99 | 107 | 119 | 123 | 85 | clade6 |
| sample_71 | 128 | 140 | 98 | 106 | 119 | 122 | 86 | clade6 |
| sample_155 | 128 | 135 | 98 | 106 | 119 | 122 | 87 | clade6 |
| sample_123 | 128 | 140 | 98 | 106 | 115 | 119 | 88 | clade6 |
| sample_130 | 128 | 137 | 95 | 104 | 119 | 119 | 89 | clade6 |
| sample_386 | 128 | 129 | 98 | 106 | 119 | 119 | 90 | clade6 |
| sample_109 | 128 | 136 | 106 | 107 | 120 | 122 | 91 | clade6 |
| sample_11 | 134 | 143 | 98 | 110 | 119 | 122 | 92 | clade6 |
| sample_316 | 131 | 144 | 98 | 104 | 116 | 119 | 93 | clade6 |
| sample_46 | 132 | 144 | 98 | 99 | 116 | 119 | 94 | clade6 |
| sample_166 | 129 | 143 | 99 | 100 | 118 | 120 | 95 | clade6 |
| sample_235 | 128 | 144 | 98 | 103 | 119 | 119 | 96 | clade6 |
| sample_201 | 129 | 143 | 96 | 98 | 119 | 119 | 97 | clade6 |
| sample_67 | 130 | 143 | 96 | 99 | 115 | 119 | 98 | clade6 |
| sample_91 | 128 | 144 | 104 | 105 | 115 | 119 | 99 | clade6 |
| sample_76 | 124 | 144 | 94 | 103 | 119 | 122 | 100 | clade6 |
| sample_86 | 128 | 154 | 98 | 106 | 122 | 122 | 101 | clade6 |
| sample_407 | 128 | 144 | 104 | 106 | 119 | 131 | 102 | clade6 |
| sample_64 | 128 | 144 | 104 | 106 | 120 | 131 | 103 | clade6 |
| sample_242 | 129 | 147 | 98 | 98 | 119 | 131 | 104 | clade6 |
| sample_291 | 130 | 147 | 95 | 95 | 119 | 131 | 105 | clade6 |
| sample_112 | 135 | 138 | 98 | 98 | 122 | 131 | 106 | clade6 |
| sample_121 | 142 | 142 | 95 | 95 | 122 | 122 | 107 | clade6 |
| sample_156 | 142 | 142 | 95 | 96 | 122 | 122 | 108 | clade6 |
| sample_98 | 143 | 143 | 96 | 96 | 117 | 121 | 109 | clade6 |
| sample_192 | 135 | 143 | 96 | 99 | 119 | 122 | 110 | clade6 |
| sample_238 | 135 | 142 | 96 | 96 | 119 | 122 | 111 | clade6 |
| sample_332 | 135 | 143 | 95 | 98 | 122 | 122 | 112 | clade6 |
| sample_150 | 135 | 141 | 98 | 99 | 117 | 122 | 113 | clade6 |
| sample_115 | 135 | 137 | 95 | 95 | 116 | 119 | 114 | clade6 |
| sample_308 | 152 | 152 | 95 | 95 | 116 | 122 | 115 | clade7 |
| sample_260 | 144 | 144 | 103 | 107 | 119 | 137 | 116 | clade6 |
| sample_199 | 106 | 113 | 99 | 113 | 119 | 122 | 117 | clade7 |
| sample_288 | 93 | 124 | 99 | 104 | 119 | 122 | 118 | clade7 |
| sample_168 | 93 | 93 | 98 | 111 | 121 | 121 | 119 | clade7 |
| sample_26 | 130 | 169 | 99 | 107 | 119 | 131 | 120 | clade7 |
| sample_310 | 135 | 161 | 99 | 107 | 119 | 131 | 121 | clade7 |
| sample_49 | 128 | 169 | 96 | 113 | 122 | 131 | 122 | clade7 |
| sample_89 | 127 | 169 | 96 | 99 | 119 | 122 | 123 | clade7 |
| sample_169 | 169 | 169 | 96 | 113 | 119 | 122 | 124 | clade7 |
| sample_252 | 169 | 169 | 96 | 107 | 119 | 122 | 125 | clade7 |
| sample_80 | 169 | 169 | 105 | 113 | 119 | 122 | 126 | clade7 |
| sample_95 | 144 | 175 | 104 | 113 | 119 | 122 | 127 | clade7 |
| sample_170 | 113 | 128 | 95 | 99 | 102 | 109 | 128 | clade7 |
| sample_415 | 92 | 169 | 96 | 99 | 122 | 122 | 129 | clade7 |

Note: Nr. : number.
